# miRNA-200c-3p targets talin-1 to regulate integrin-mediated cell adhesion

**DOI:** 10.1038/s41598-021-01143-3

**Published:** 2021-11-03

**Authors:** Gideon Obeng, Eun Jeong Park, Michael G. Appiah, Eiji Kawamoto, Arong Gaowa, Motomu Shimaoka

**Affiliations:** 1 Department of Molecular Pathobiology and Cell Adhesion Biology, Mie University Graduate School of Medicine, Tsu, Mie 514-8507 Japan; 2 Department of Emergency and Disaster Medicine, Mie University Graduate School of Medicine, Tsu, Mie 514-8507 Japan

**Keywords:** Cell biology, Immunology, Molecular biology

## Abstract

The ability of integrins on the cell surface to mediate cell adhesion to the extracellular matrix ligands is regulated by intracellular signaling cascades. During this signaling process, the talin (TLN) recruited to integrin cytoplasmic tails plays the critical role of the major adaptor protein to trigger integrin activation. Thus, intracellular levels of TLN are thought to determine integrin-mediated cellular functions. However, the epigenetic regulation of TLN expression and consequent modulation of integrin activation remain to be elucidated. Bioinformatics analysis led us to consider miR-200c-3p as a *TLN1*-targeting miRNA. To test this, we have generated miR-200c-3p-overexpressing and miR-200c-3p-underexpressing  cell lines, including HEK293T, HCT116, and LNCaP cells. Overexpression of miR-200c-3p resulted in a remarkable decrease in the expression of *TLN1*, which was associated with the suppression of integrin-mediated cell adhesion to fibronectin. In contrast, the reduction in endogenous miR-200c-3p levels led to increased expression of *TLN1* and enhanced cell adhesion to fibronectin and focal adhesion plaques formation. Moreover, miR-200c-3p was found to target *TLN1* by binding to its 3′-untranslated region (UTR). Taken together, our data indicate that miR-200c-3p contributes to the regulation of integrin activation and cell adhesion via the targeting of *TLN1*.

## Introduction

Integrins are the family of cell adhesion molecules that mediate cell adhesion in a wide-range of biological contexts^1^. The adhesiveness of integrins is dynamically regulated by inside-out signals, in which binding of talin to the integrin cytoplasmic domain triggers activation-dependent conformational changes^2,3^. Talin is a large intracellular protein (~ 270 kDa) critical to the regulation of cell-to-cell and cell-to-extracellular matrix (ECM) communications^4^. Composed of more than 2500 amino acid residues, and organized into multiple domains^5^, talin provides multiple binding sites for proteins involved in cell adhesion^6^. The N-terminal end of talin, also called the head, includes 4 domains (4.1, ezrin, radixin, and moesin; collectively called FERM) and is joined to the C-terminal rod by a linker^7^. The rod contains 13 α helix-rich domains capped at the end by a dimerization domain. This terminal domain ensures that talin can function as a dimer under physiological conditions^8^. In mammalian tissues, there are two talin paralogues, TLN1 and TLN2*.* Both genes (*TLN1* and *TLN2*) encode proteins that share gross identity in amino acid sequences (~ 74%) and domain structures^5^. In addition, TLN1 and TLN2 largely associate with proteins related to each other, and thus can share many cellular functions^9,10^. Nonetheless, TLN1 is predominantly expressed in most tissues and appears to be indispensable, as the deletion of this gene in mouse endothelial cells results in internal vascular haemorrhaging and subsequently death^11^.

Talin, for the most part, coordinates the assembly of multiprotein adhesion complexes that mediate biological processes such as cell migration and, ultimately, more complex phenomena including embryogenesis and tumorigenesis^6^. To facilitate these assemblages, talin initially forms a nascent complex through its intracellular interaction with the transmembrane receptor, integrin^12^, and the cytoskeletal protein, actin^13^. These interactions typically enhance the affinity of integrins to associated ligands, while also establishing a communication linkage between cells and ECM^3^. Intriguingly, these interactions may induce the unfolding of the talin rod domains, thereby providing more binding sites for other proteins such as vinculin^14^. Vinculin stabilizes the focal adhesion complex and is crucial to its maturation^14^. The importance of talin in orchestrating these multiprotein assemblages and impacting several important biological processes has been highlighted in studies with mice whose *TLN* genes were ablated^9,11^. Deletion of both *TLN1* and *TLN2* in cardiomyocytes has resulted in heart failure and consequently death, possibly due to the decrease in expression and function of costameric proteins, notably integrin β1D^9^.

MicroRNAs (miRNA or miR) are relatively short and single-stranded non-coding RNAs that post-transcriptionally regulate gene expression^15^. These molecules essentially act as guide RNAs within gene silencing complexes to induce mRNA cleavage and/or translational repression^16^. Seed sequences, which are miRNA-specific sequences spanning nucleotide positions two to eight, bind response elements within the 3′-UTR of messenger RNAs to heavily facilitate this process^17,18^. Therefore, miRNAs with similar seed sequences, and likely to have the same gene targets, have been grouped together into miRNA families^17,18^. miR-200c is a member of the miR-200 family, which also includes miR-141, miR-200a, miR-200b and miR-429^19^. These members share identical seed sequences with only a single nucleotide difference between subgroups. Notably, this family regulates several important biological processes, most notably epithelial-to-mesenchymal transition (EMT), which is a critical process in cancer metastasis^19,20^. Expression of these miRNAs is usually upregulated in cancerous epithelial cells and these miRNAs are known to target various transcriptional repressors of E-cadherin, especially the zinc finger E-box binding homeobox 1 (ZEB1) family of transcriptional factors^19–21^, as well as other related proteins to modulate EMT^22^.

Given the fundamental importance of integrin-mediated cell adhesion to EMT^23–25^, we have performed a bioinformatics analysis using miRDB (www.mirdb.org) and TargetScan (http://www.targetscan.org/vert_72/) in order to identify targets of miR-200 that might regulate integrin-activation. Our analyses identified several miR-200 family members, including miR-200b-3p, miR-200c-3p, and miR-429 that were predicted to target talin genes. Of those miR-200 family members, the role of miR-200c-3p in regulating cellular adhesion or migration has been most extensively studied^26–31^. Thus, we strategically chose to focus on miR-200c-3p and asked whether TLN1 was epigenetically modified by this microRNA. In addition, we sought to determine whether by regulating the expression of *TLN1*, miR-200c-3p could also impact integrin-mediated cell adhesion. Here we have shown that overexpression of miR-200c-3p decreased the expression of *TLN1* and subsequently downregulated integrin-mediated cell adhesion, whereas knockdown of miR-200c-3p increased *TLN1* expression and upregulated the integrin-mediated binding to fibronectin. Our study suggests that cellular levels of miR-200c-3p govern integrin-mediated cell adhesion via targeting *TLN1*.

## Results

### TLN1 expression is altered significantly by the manipulations of miR-200c-3p levels

**Figure 1:**
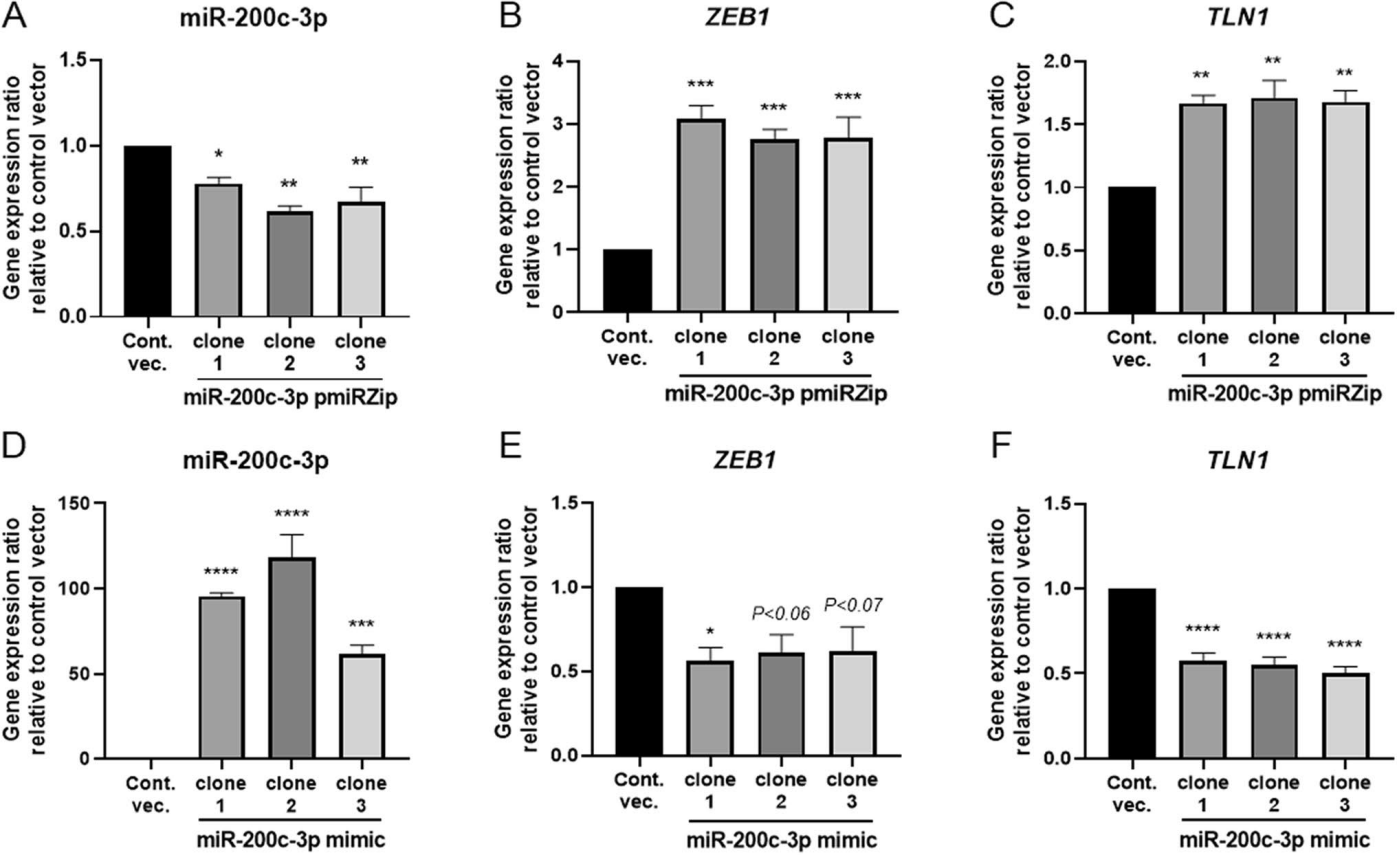
Regulation of miR-200c-3p, *ZEB1*, and *TLN1* expression in HEK293T cells. HEK293T cells were stably transduced with miR-200c-3p pmiRZip to down-regulate miR-200c-3p expression (**A**–**C**), miR-200c-3p pre-miR (miR-200c-3p mimic) to upregulate miR-200c-3p expression (**D**–**F**), or an empty vector (control), using lentivector systems. The expression levels of miR-200c-3p (**A**, **D**), *ZEB1* (**B**, **E**), and *TLN1* (**C**, **F**) in three representative clones per group were measured using RT-qPCR. The control values in each panel were normalized to 1. All assays were performed in triplicates and the experiments were repeated three times. Data are expressed as the mean ± standard errors of the mean (SEM) relative to the control. *P < 0.05; **P < 0.01; ***P < 0.001; and ****P < 0.0001.

To test the hypothesis that miR-200c-3p is involved in the epigenetic regulation of integrin activation triggered by talin, we transduced HEK293T cells with either miR-200c-3p-knockdown vector pmiRZip (miR-200c-3p pmiRZip) to downregulate miR-200c-3p expression, or with pre-miR-200c-3p (miR-200c-3p mimic) to upregulate miR-200c-3p expression. The miR-200c-3p pmiRZip transduced clones exhibited an approximately 25% decrease in miR-200c-3p expression (Fig. 1A). The clones transduced with miR-200c-3p mimic exhibited greater than 100 times increase in miR-200c-3p levels (Fig. 1D). As ZEB1 was previously shown to be a target of miR-200c-3p^20,21,32,33^, we next examined the expression levels of *ZEB1* in different clones. As expected, we confirmed that *ZEB1* expression was increased in the miR-200c-3p pmiRZip-transduced cells, whereas *ZEB1* expression was suppressed in the miR-200c-3p mimic-transduced cells (Fig. 1B,E). Then, we proceeded to investigate how *TLN1* expression was affected by miR-200c-3p levels. The miR-200c-3p pmiRZip transduced clones exhibited a marked increase in *TLN1* expression, while the miR-200c-3p mimic-transduced cells showed a significant reduction in *TLN1* expression (Fig. 1C,F). In addition, miR-200c-3p mimic and miR-200c-3p pmiRZip transduced clones did not show any changes in the expression of interleukin-4 (IL-4) and mucosal address in cell adhesion molecule-1 (MAdCAM-1), both of which are not predicted as miR-200c-3p targets and, thereby, used as negative controls (Fig. S2A–D).

In addition to TLN1, the bioinformatics analysis using TargetScan predicted that TLN2 was among the miR-200c-3p’s targets. Other focal-adhesion proteins such as vinculin, paxillin, integrin-linked kinase (ILK) and focal adhesion kinase (FAK) were not predicted as miR-200c-3p’s targets. Endogenous levels of *TLN2* were much lower in HEK293T cells than those of *TLN1* (Fig. S3A), although the expression of *TLN2* was also affected by miR-200c-3p (Fig. S3B,C). Thus, in the present study we decided to focus only on the predominant talin gene, *TLN1* .

To substantiate the findings that *TLN1* expression is inversely correlated with the levels of miR-200c-3p in HEK293T cells, we expanded our analysis to the cell lines HCT116 (a human colorectal cancer cell line) and LNCaP (a human prostate cancer cell line), which express high levels of miR-200c-3p^34,35^. HCT116 and LNCaP cells transduced with miR-200c-3p pmiRZip were subjected to the analysis for *TLN1* and *ZEB1* expression. We confirmed in both miR-200c-3p pmiRZip-transduced HCT116 and LNCaP cells a significant decrease in miR-200c-3p expression (Fig. S4) and concomitant increase in the *ZEB1* and *TLN1* expression (Fig. S5), compared to control cells. Therefore, miR-200c-3p-mediated downregulation of *TLN1* shown in HEK293T cells (Fig. 1) was also substantiated in cells that express relatively higher levels of endogenous miR-200c-3p.

**Figure 2:**
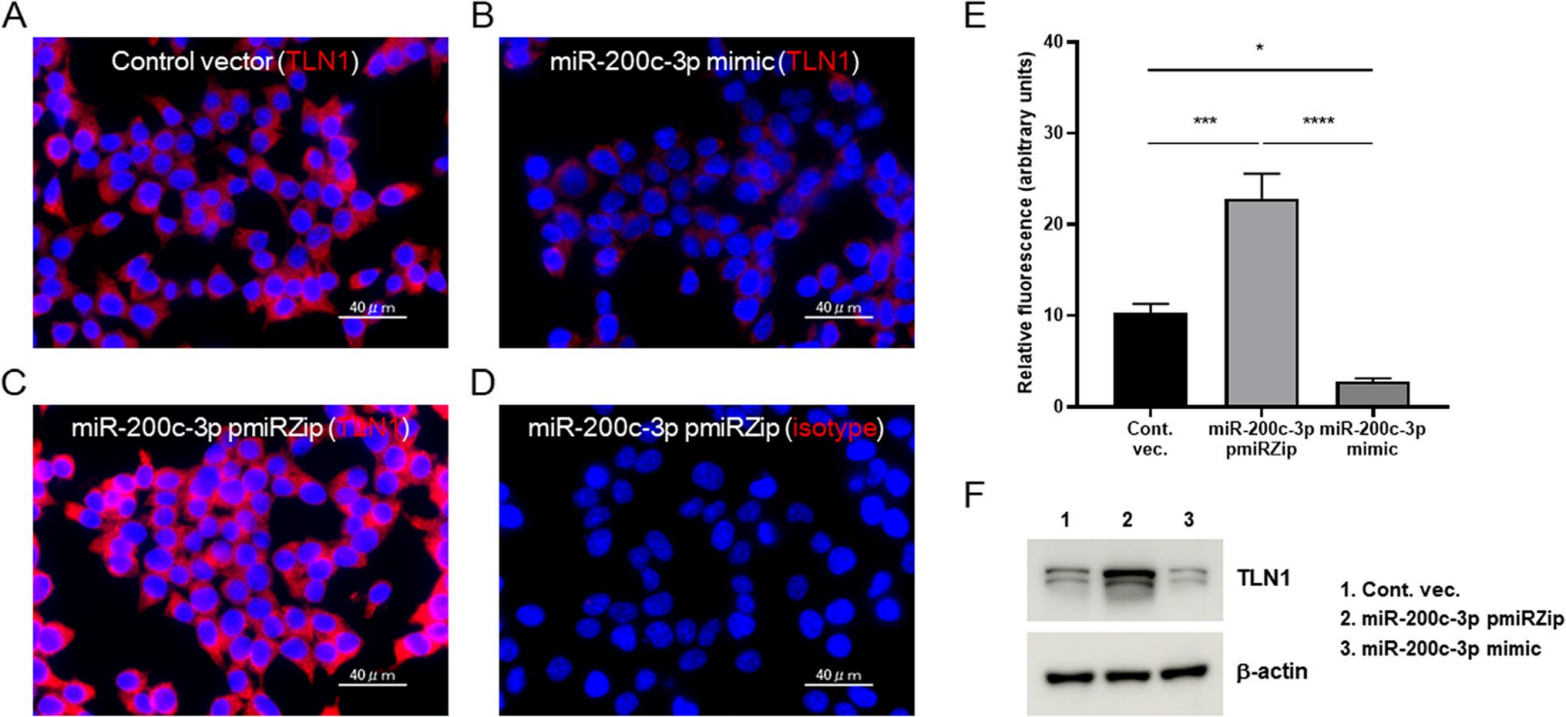
miR-200c-3p downregulates the expression of TLN1 protein in cells. (**A**–**D**) HEK293T cells stably transduced with control vector (**A**), miR-200c-3p pmiRZip (**B**), or mimic/pre-miR (**C**) were fixed and stained with anti-TLN1 antibody and an allophycocyanin-tagged secondary antibody (red). Nuclear staining was done with DAPI (blue). To gauge the backgroud level of staining (**D**), an isotype-matched antibody (mouse IgG1) was used to stain HEK293T cells transduced with miR-200c-3p pmiRZip. All images were acquired using identical capture and processing protocols. Similar results were seen in three separate experiments. (**E**) The cellular levels of TLN1 expressions were quantified by analyzing relative fluorescence intensities (see “Methods” for details). Data are expressed as the mean ± standard errors of the mean (SEM) relative to the control. *P < 0.05; ***P < 0.001; and ****P < 0.0001. (**F**) Immunoblot analysis shows levels of TLN1 expressed in the cells stably transduced with miR-200c-3p pmiRZip, miR-200c-3p mimic, or control vector and β-actin was used as an internal control. The original full blots are shown in Supplementary Fig. S1. Scale bar, 40 µm (**A**–**D**). *Cont. vec.* control vector.

We next performed immunofluorescence analysis to ask whether miR-200c-3p also triggers a reduction in TLN1 expression in HEK293T cells. The cells were stained with anti-TLN1 antibody and an allophycocyanin-tagged secondary antibody and analyzed for levels of TLN1 protein. As shown in the representative images in Fig. 2, the cells transduced with miR-200c mimic vector exhibited a drastic reduction in TLN1 expression (Fig. 2B), compared to those transduced with control vector (Fig. 2A). In contrast, the cells transduced with miR-200c-3p pmiRZip vector showed a remarkable increase in TLN1 (Fig. 2C). As a control to gauge a background signal level, the miR-200c-3p pmiRZip-transduced cells stained with isotype control (mouse IgG1) hardly showed signals (Fig. 2D). A quantitative analysis of multiple images confirmed that talin-1 protein significantly increased in the miR-200c-3p pmiRZip-transduced cells and decreased in miR-200c-3p mimic-transduced cells (Fig. 2E), which was further supported by the immunoblot analysis (Fig. 2F). Taken together, miR-200c-3p-mediated downregulation of TLN1 expression as shown at both mRNA and protein levels, thereby suggesting that miR-200c-3p regulates TLN1 expressions.

### miR-200c-3p targets the 3′-UTR of *TLN1*

**Figure 3:**
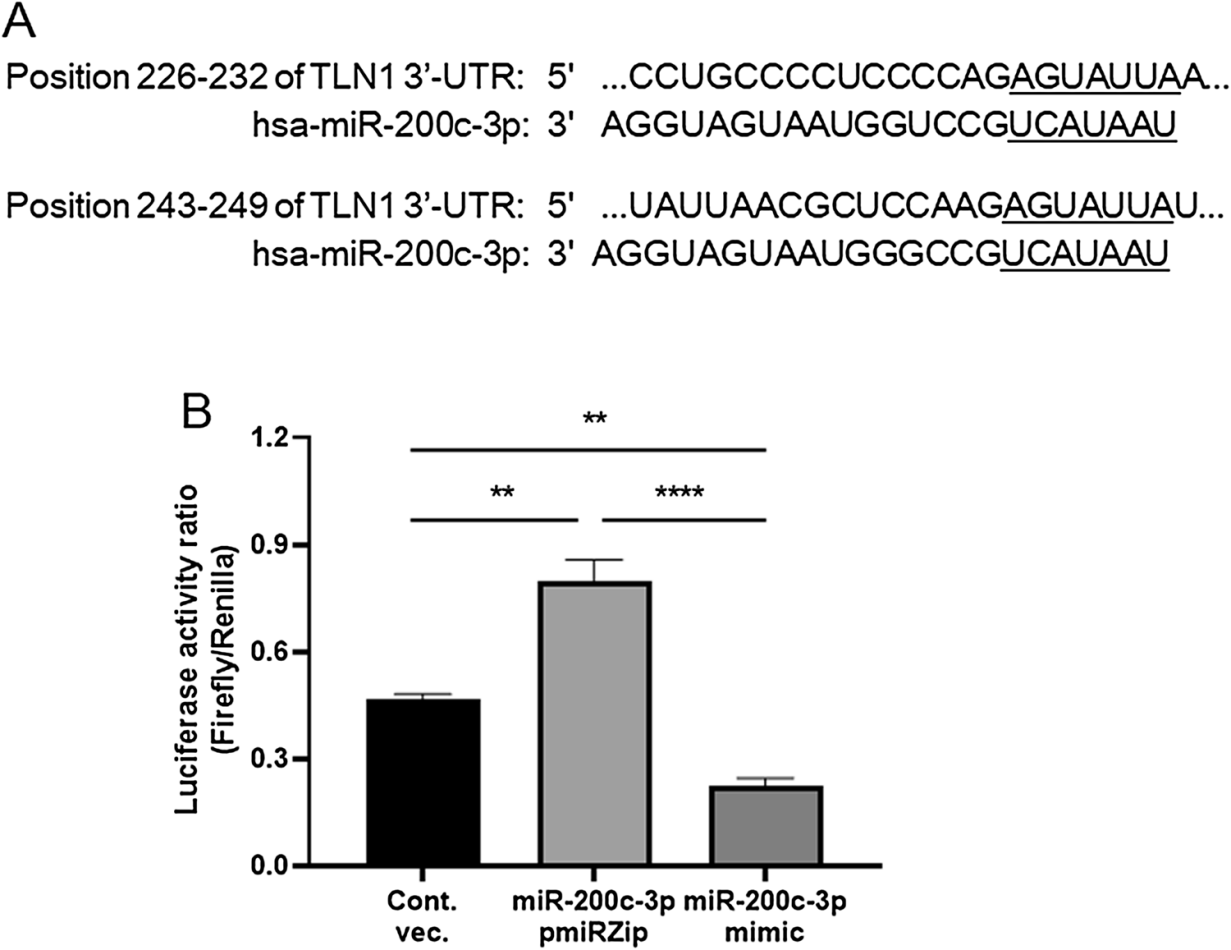
miR-200c-3p targets the 3′-UTR of *TLN1*. (**A**) Schematic representation of the two conserved binding sites for miRNA-200c-3p on *TLN1* 3′-UTR and their potential interactions with miRNA-200c-3p seed sequences. The miR-200c-3p seed sequences and their target sequences are underlined. (**B**) Luciferase reporter activities were measured, 48 h after HEK293T cells were co-transfected with the *TLN1*-3′-UTR-luciferase construct and miR-200c-3p pmiRZip, miR-200c-3p mimic, or control vector (Cont. vec.). All assays were performed in triplicates and experiments were repeated three times. Data are expressed as the mean ± standard errors of the mean (SEM) relative to the control vector. **P < 0.01; and ****P < 0.0001.

To study the epigenetic mechanism by which miR-200c-3p regulates TLN1 expression, we investigated the interactions of miR-200c-3p with 3*′*-UTR of *TLN1* mRNA. An in silico analysis predicted that there are two miR-200c-3p binding sites within 3*′*-UTR of *TLN1* (www.mirdb.org) (Fig. 3A). To demonstrate that miR-200c-3p binds 3*′*-UTR of the *TLN1*, thereby regulating its expression, a dual-luciferase reporter assay system was employed. We co-transfected HEK293T cells with either miRNA-200c-3p pmiRZip construct or mimic/pre-miR construct and a 3*′*-UTR (*TLN1*) firefly luciferase construct that contained the two miR-200c-3p binding sites, designated as miR-200c-3p pmiRZip and mimic, respectively. Control cells were co-transfected with the empty lentivector and the same firefly luciferase construct as mentioned above, termed control vector. Three of all transfectants (control vector, miR-200c-3p pmiRZip, and miR-200c-3p mimic) were further transfected with Renilla luciferase reporter vector to normalize the firefly luciferase activity. Using this dual-reporter assay system, we measured luciferase reporter activities. Compared to those in control-vector transfected cells, the *TLN1* 3′-UTR-specific firefly luciferase activities were significantly increased in the miR-200c-3p pmiRZip-transfected cells, and inversely, decreased in the miR-200c-3p mimic-transfected cells (Fig. 3B). Collectively, these results support a possible mechanism that miR-200c-3p regulates *TLN1* expression by directly targeting its 3*′*-UTR.

### miR-200c-3p suppresses cellular binding to fibronectin possibly by reducing TLN1

**Figure 4:**
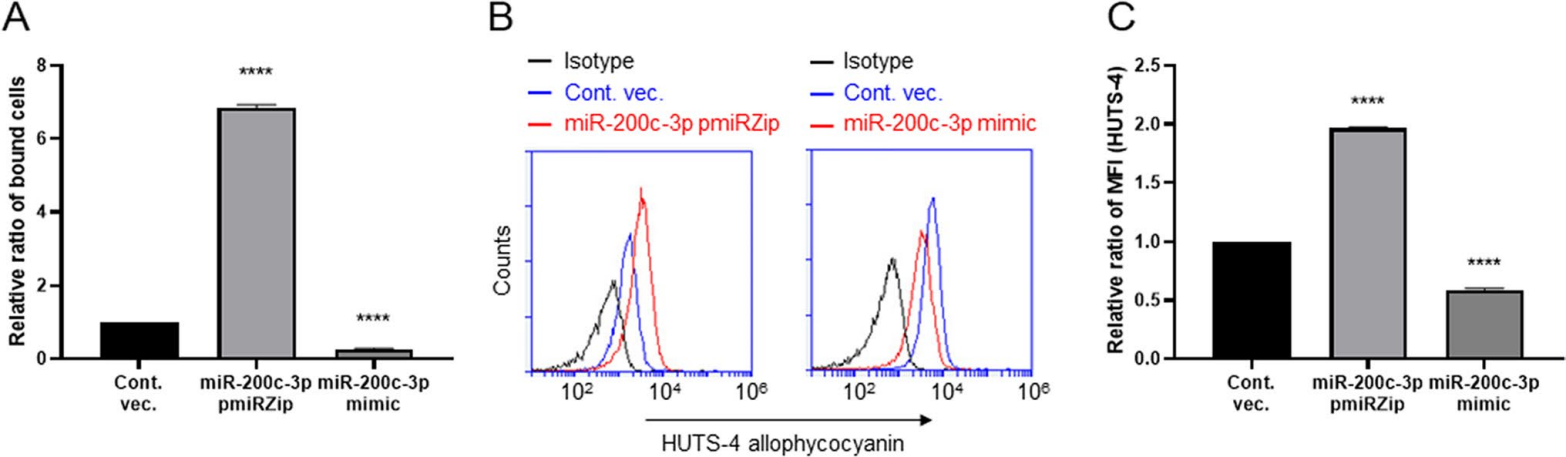
miR-200c-3p negatively regulates cell binding to fibronectin. (**A**) The cell binding to fibronectin coated on V-bottom wells was measured and compared between the HEK293T cells transduced with miR-200c-3p pmiRZip, miR-200c-3p mimic, or empty vector (control). The percentages of bound cells were determined as described in the Methods section, and the relative ratios to control vector-transduced cells are shown. (**B**, **C**) The cells were incubated with HUTS-4 (mAb that interacts with the activation-dependent epitope of β1 integrin) or mouse isotype control antibody (IgG2b). Bound mAbs were detected with allophycocyanin-conjugated secondary antibody and analyzed using flow cytometry. Representative FACS histograms from three independent experiments are shown (**B**). MFI values for HUTS-4 mAb were determined and relative levels of HUTS-4 binding to control vector-transduced cells were compared (**C**). (**A**, **C**) Data are expressed as the mean ± standard errors of the mean (SEM) relative to the control vector (Cont. vec.). All assays were performed in at least triplicates and the experiments were repeated three times. *MFI*, mean fluorescence intensity. ****P < 0.0001.

TLN is known to be the major adaptor protein that is indispensable for the final common step in integrin-mediated cellular adhesion^3,6,36^. We hypothesized that intracellular levels of miR-200c-3p determine integrin-mediated cell adhesion by modulating TLN1 expression. To test this, we conducted a cell-adhesion assay using miR-200c-3p pmiRZip and mimic clones. After coating V-bottom 96-well plates with fibronectin, a major ECM integrin ligand for integrin α5β1^37^, we measured the strength of cell adhesion in the presence of 1 mM CaCl_2_ and 1 mM MgCl_2_. The miR-200c-3p pmiRZip-transduced cells exhibited a remarkable increase in binding to fibronectin, while a clone of miR-200c-3p mimic-transduced cells displayed significantly lower fibronectin adhesion, compared to cells transduced using vector control (Fig. 4A). Furthermore, we have shown that both HCT116 and LNCaP cells transduced with miR-200c-3p pmiRZip also exhibited a significant increase in binding to fibronectin (Fig. S6), confirming the results from analysis with HEK293T cells (Fig. 4A). To substantiate the results obtained with the V-bottom plate cell adhesion assay, in which unbound cells were removed by the centrifugal force, we performed a conventional adhesion assay using a flat-bottom plate, which unbound cells were removed by the washing procedure that generated turbulent flow. The conventional cell adhesion assay recapitulated the results in HEK293T cells (Fig. S7A) and, thereby, showed that integrin α5β1 mediated cell adhesion to fibronectin (Fig. S7B).

Direct association of vinculin with talin and actin triggers the formation of focal adhesion via integrin clustering, which governs cell adhesion and migration^38^. As talin is the essential component of focal adhesion^39^, we have studied how miR-200c-3p affected the formation of focal adhesion plaques. To this end, we performed immunofluorescence imaging of vinculin and actin in the miR-200c-3p pmiRZip-transduced HEK293T cells. Compared with control vector-transduced cells (Fig. S8A), miR-200c-3p pmiRZip-transduced cells exhibited a remarkable increase in the zones of association of vinculin with actin (Fig. S8B). Thus, these results suggest that downregulation of miR-200c-3p promotes the formation of focal adhesion plaques.

miR-200c-3p-mediated modulation of TLN1 levels is thought to affect cell adhesion to fibronectin by impacting on the activation-dependent conformational changes of integrin α5β1^2,3^. We thus sought to examine how miR-200c-3p loss-of-function (knockdown) and gain-of-function/overexpression (mimic) would affect integrin conformations probed by an activation-dependent epitope. To examine whether miR-200c-3p expression altered integrin’s activation we employed antibody HUTS-4, which preferentially binds to an integrin epitope that is exposed only when β1 integrin is in an active state^40^ in combination with flow cytometry. As shown in Fig. 4B,C, miR-200c-3p pmiRZip- and miR-200c-3p mimic-transduced cells exhibited a significant increase and reduction, respectively, in binding of HUTS-4, compared with control vector-transduced cells. Overall, these data underscore that miR-200c-3p modifies talin-1-mediated integrin activation pathways that culminate to the epigenetic regulation of integrin-mediated cell adhesion.

## Discussion

A proper balance in the activation and deactivation of integrins is critical for up- and down-regulating cell adhesion to ECM, which constitute some of the basic steps for various biological and pathological processes^41,42^. Integrin activation is achieved by inside-out and intracellular signaling that culminates in the association of multiple adaptor proteins to integrin cytoplasmic tails. TLN, composed of TLN1 and TLN2, is a main adaptor molecule essential to a final common step in integrin activation. Accordingly, it is of great importance to understand the molecular mechanisms by which the expression and function of TLN is regulated by endogenous molecules such as miRNAs. Here we have demonstrated that miR-200c-3p plays the important role in the epigenetic regulation of *TLN1*, thereby potentially participating in the fine-tuning of the integrin activation and conformational regulation.

miR-200c-3p, which belongs to the miR-200 family, is known to be a functionally versatile miRNA that negatively regulates EMT by targeting *ZEB1*^20,22,43^, which was also confirmed in our study (Fig. 1B,E). This miRNA has also been shown to modulate cancer stem-cell heterogeneity by targeting home domain-interacting protein kinase 1 (*HIPK1*)^30^. In addition, miR-200c-3p suppresses tumor-cell growth and invasion by targeting B-cell-specific Moloney murine leukemia virus insertion site 1 (*BMI1*) and E2F transcription factor 3 (*E2F3*)^29^, both of which have similarly been targets of miR-128-1^44^ and miR-429 (a member of the miR-200 family)^45^. Thus, miR-200c-3p is thought to comprise the miRNA-target gene regulatory network that interferes with epithelial-carcinoma invasiveness. Hoefert et al. have demonstrated that miR-200 family members, including miR-200c-3p, regulate epithelial cell binding and growth during normal hair morphogenesis by cooperatively targeting multiple genes such as cyclin G2 (*CCNG2*), cyclin D2 (*CCND2*), cofilin-2 (*CFL2*), and snail family transcriptional repressor 2 (*SNAI2*)^46^. In addition, miR-200c-3p has been also shown to downregulate NADPH oxidase activator termed Noxa which is known to mediate apoptosis^47^, to thereby contribute to attenuating pro-apoptotic pathway of non-malignant cells^48^. Zhang et al., have reported that by targeting Notch1, miR-200c suppressed endothelial proliferation and metabolism under hyperglycaemic state^49^.

Kindlin-2 is structurally and functionally related to the TLN^50^, and transcripts of kindlin-2 have proven to be a direct target of miR-200b for the modulation of EMT in breast cancer cell metastases^51^. As mentioned earlier, bioinformatics analysis has shown that miR-200 family members can potentially modulate *TLN1* expression. Despite scant evidence for any direct interaction between these miR-200 members and *TLN1* thus far, some other miRNAs such as miR-9, miR-124, and miR-330 have been previously reported to target *TLN1* in order to regulate its expression in cancer cell. Several miRNAs such as miR-9, miR-124, and miR-330 have been previously reported to target *TLN1* to regulate its expression. Treatment with miR-9 for ovarian serous carcinoma cells suppressed their migratory and invasive capabilities by targeting *TLN1* and downregulating its expression^52^. Likewise, miR-124 was effective at targeting *TLN1* and interfering with dynamic activity of prostate cancer cells^53^. In addition, miR-330 has been shown to regulate the proliferative and angiogenic properties of hepatocellular carcinoma cells by targeting *TLN1*^54^. However, miRNA regulation of *TLN1* expression, which leads to the modification of integrin-mediated cell adhesion, remains to be elucidated. Thus, in this study we sought to identify a new *TLN1*-targeting miRNA and examine the effects of its altered levels on cell adhesiveness.

miRNAs and their targets participate to regulatory network implicated in cellular functions such as invasion through a coordinated role played by multiple components^55^. It is unlikely that single-gene downregulation by certain miRNA independently impinge on modifying cell adhesion. Indeed, miR-200c-3p may have several-hundred targets^56^. Therefore, a possibility cannot be ruled out that the reduced cell adhesion occurred by miR-200c-3p-mediated TLN1 downregulation is integrated with the concomitant expressional decrease in other targets such as kindlin-2^57^ or E2F transcription factor 3 (E2F3)^29^.

Our current results support the possibility that miR-200c-3p directly targets TLN1 and regulates its expression. There is currently no evidence supporting or refuting the possibility that TLN1 is transcriptionally regulated by ZEB1, an EMT transcription factor targeted by miR-200c-3p. However, given the complexity and interdependence of the miRNA regulatory network^58^, further investigations in the future may reveal any secondary effects of miR-200c-3p regulatory network that could impact on regulating TLN1 expression.

With regards to current finding that miR-200c-3p targets *TLN1* (Fig. 3B), miR-200c-3p-dependent TLN1 up- and down-regulation modified integrin-induced cell adhesion to fibronectin (Fig. 4A). These results support the notes that upregulation of TLN1 itself plays roles in activating integrins as a final trigger^3,36,59^. Notably, the TLN1 upregulated by a reduction in the intracellular miR-200c-3p was capable of converting integrin’s conformation to the high-affinity state. Based on the experiments using HUTS-4 specific for the active β1 integrin conformation^40^, the reduction of miR-200c-3p in the pmiRZip-transduced cells has enhanced the levels of β1-integrin activation, compared to control cells; and vice versa in case of the miR-200c-3p mimic-transduced cells (Fig. S7). Thus, TLN1-mediated regulations of integrin conformation and function may rely, at least partly, on cellular levels of miR-200c-3p. In agreement with these notes, it is intriguing to corroborate further roles played by miR-200c-3p in regulating β1 integrin-driven outside-in signaling in near future.

**Figure 5:**
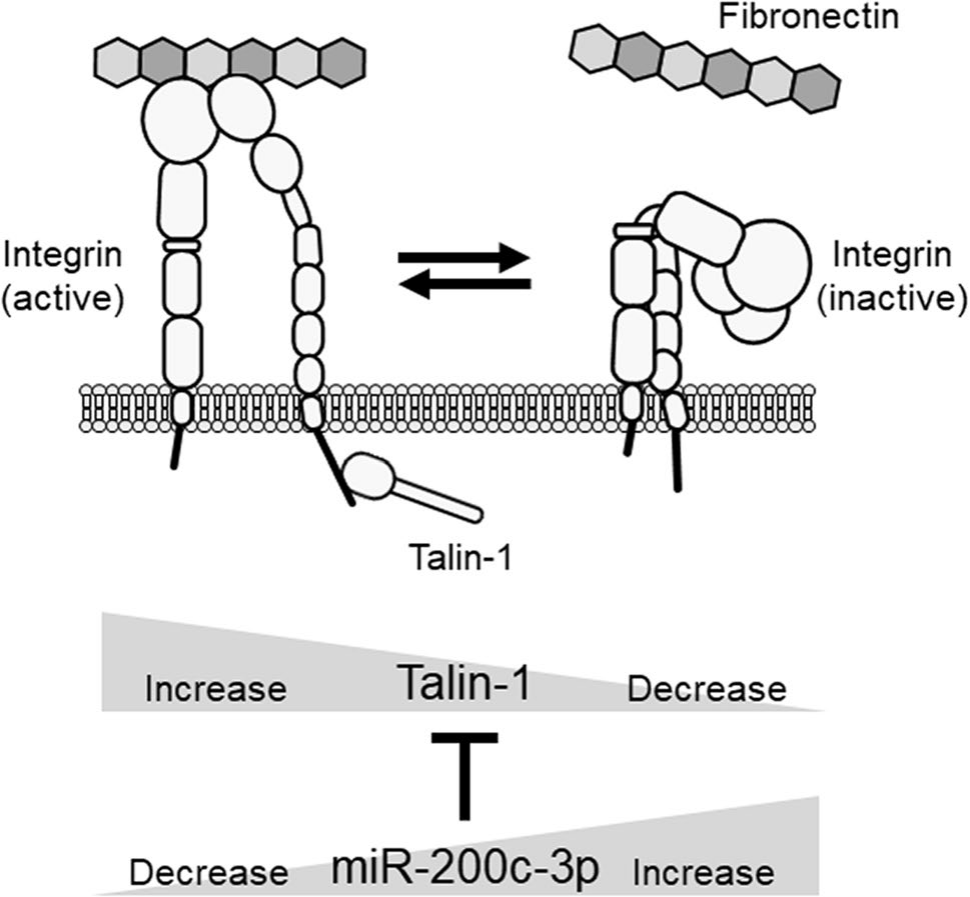
A schematic model for the regulatory role played by the miR-200c-3p-mediated *TLN1* alteration underlying integrin-mediated cell adhesion. Talin-1 (TLN1) binding to the cytoplasmic tail activates integrins to induce cell adhesion to ligands such as fibronectin. Downregulation of talin-1 by elevated miR-200c-3p suppresses integrin activation and results in decreased cell adhesion to the ligand. By contrast, upregulation of talin-1 by reduced miR-200c-3p levels enhances integrin-mediated cell adhesion.

In conclusion, we have demonstrated that miR-200c-3p targets *TLN1*. In accordance with this finding, overexpression of miR-200c-3p led to reduced cell adhesiveness, via *TLN1* downregulation, to fibronectin, which is an abundant ECM ligand for integrins such as α5β1 and αV classes^37,60^. Moreover, abrogation of this miRNA resulted in increased cell binding and the formation of focal adhesion plaques. Figure 5 illustrates this proposed model, in which an miR-200c-3p-mediated change in TLN1 levels regulated integrin activation and subsequent cell adhesion to fibronectin. Based on our current findings, it would be worthwhile to further examine the effects of *TLN1* targeting by miR-200c-3p on the functionality of different integrins (e.g., αLβ2, α4β1, or α4β7) that adhere to their cognate ligands (e.g., intercellular adhesion molecule 1, ICAM-1; vascular cell adhesion molecule 1, VCAM-1; or mucosal address in cell adhesion molecule 1, MAdCAM-1, respectively) in different cell types including immune cells.

## Methods

### Cell culture

HEK293T, HEK293TN, HCT116, and LNCaP cell lines were obtained from American Type Culture Collection (ATCC) (Manassas, VA, USA) and System Biosciences (Palo Alto, CA, USA), respectively, and were cultured in Dulbecco’s Modified Eagle Medium (DMEM) (Nacalai, Kyoto, Japan) supplemented with 4 mM l-glutamine (Nacalai), 10% fetal bovine serum (FBS) (Equitech-Bio, Kerrville, TX, USA) and penicillin/streptomycin (Nacalai) according to the manufacturer’s instructions.

### Generation of cells stably expressing miR-200c-3p mimic/pre-miR and antagomir

The lentivectors pMIRNA1 and pmiRZip (System Biosciences) containing miR-200c mimics and antagomirs, respectively, as well as control empty lentivectors (System Biosciences) were used to generate the HEK293T cell transductants for each gene. In brief, we first transduced HEK293TN cells with the aforementioned lentivectors by using PureFection reagent (System Biosciences) to package the viral particles, according to the manufacturer’s instructions. HEK293T, HCT116, and LNCaP cells were seeded at 3 × 10^5^ cells/ml in 6-well plates and allowed to grow until approximately 60% confluency. The cells were then transduced with lentiviral particles for either miRNA-200c-3p mimics or pmiRZip. Control cells were transduced with empty lentiviral particles. To enhance the transduction efficiency, polybrene (Nacalai) was added to culture medium at a concentration of 8 µg/ml. After 24 h, the culture medium was replaced with fresh medium containing 1 µg/ml puromycin (InvivoGen, San Diego, CA, USA). Cells were further cultured for 48 h to select those cells resistant to puromycin. Single-cell clones of HEK293T were generated using the the method of limiting dilution. Briefly, the cells (5 per ml) were seeded in 96-well plates and cultured in the presence of 1 µg/ml puromycin for 7–14 days. Wells with single-cell colonies were isolated and expanded further for subsequent experiments.

### Reverse transcription and quantitative polymerase chain reaction (RT-qPCR)

Total RNA was extracted from cells using Trizol reagent (Thermo Fisher Scientific, Waltham, MA, USA) and measured for the concentration using a NanoDrop2000 spectrophotometer (Thermo Fisher Scientific). Approximately 1 µg of RNA was used to perform RT in a reaction by using a Prime Script RT Kit (Takara Bio, Shiga, Japan) for mRNA or a Mir-X miRNA First-Strand Synthesis Kit (Takara Bio) for miRNA according to the manufacturer’s instructions. Then, qPCR was conducted by using a PowerUp SYBR Master Mix (Applied Biosystems, Forster City, CA, USA) and a StepOne Real-Time PCR device (Applied Biosystems). *U6* and *β-actin* were used as reference genes to analyze the relative expressions of miRNA and mRNAs, respectively. For the miR-200c-3p, a universal primer (Thermo Fisher Scientific) was used as a reverse primer. Primer sequences (5′ → 3′) used in the qPCR were as follows: *U6*, GCGCGTCGTGAAGCGTTC (forward) and GTGCAGGGTCCGAGGT (reverse); *hsa-miR-200c-3p*, TAATACTGCCGGGTAATGATGGA; *β-actin*, GATGCAGAAAGGAGATCACTG (forward) and CGATCCACACGGAGTACTTG (reverse); *TLN1*, TCTCCCAAAATGCCAAGAAC (forward) and TGGCTATTGGGGTCAGAGAC (reverse); *ZEB1*, GCCAATAAGCAAACGATTCTG (forward) and TTTGGCTGGATCACTTTCAAG (reverse); *IL-4*, ACTTTGAACAGCCTCACAGAG (forward) and TTGGAGGCAGCAAAGATGTC (reverse); and *MAdCAM-1*, CTGTACGGCCCACAAAGTCA (forward) and TCTGTCACCCTGAACAGCAC (reverse). Relative expression to the reference gene using comparative threshold (CT) values was normalized to controls and shown as 2^−∆∆CT^ unless otherwise specified.

### Immunofluorescence analysis

The 10 µg/ml fibronectin (Sigma-Aldrich, St. Louis, MO, USA) was coated onto 18-mm glass coverslips (Instruments, Hamden, CT, USA) for 1 h at 37 °C^61^. HEK293T cell clones in the culture medium (see Sect. 2.1. above) were cultured on the coverslips for 16 h and then fixed with ice-cold methanol for 1 min as described previously^62^. Cells were then blocked in 1% bovine serum albumin (BSA) (Sigma-Aldrich) for 1 h at 37 °C and incubated with 7 µg/ml of mouse anti-human TLN1 monoclonal antibody (mAb) (97H6, Bio-Rad, Hercules, CA, USA) or mouse IgG1 (isotype) (Biolegend) for 16 h at 4 °C. Then, the cells were incubated with allophycocyanin-labeled anti-mouse IgG antibody (Biolegend, San Diego, CA, USA) at room temperature for 30 min and counterstained with 4′,6-diamidino-2-phenylindole (DAPI) for nuclear staining. In some experiments, the cells were stained for vinculin and phalloidin (actin) by using actin cytoskeleton/focal adhesion staining kit (Merck, Darmstadt, Germany) according to the manufacturer’s instructions. The coverslips were mounted on slide glasses using an Aqueous Mounting Medium (Abcam, Cambridge, UK). Images were acquired on a fluorescence microscope (BZ-X710, Keyence, Osaka, Japan).

To quantify the expression of TLN1, images were analyzed using ImageJ (v1.53f) (NIH, Bethesda, MD, USA) as previously described^63^. In brief, regions of interest were outlined using a drawing tool and mean fluorescence was measured. Adjacent background readings were also performed. Corrected total cellular fluorescence (CTCF) was calculated by a formula of {integrated density – (area of selected cell × mean fluorescence of background readings)}. Several images per each group were used to acquire CTCF which was shown as mean values of the relative fluorescence (arbitrary units). Bar graphs and statistical analysis {one-way analysis of variance (ANOVA)} were done using Prism 8 (GraphPad, San Diego, CA, USA).

### Immunoblot analysis

Immunoblot analysis was done as previously described with some modifications^64^. In brief, cells were lysed in RIPA buffer (Nacalai), and proteins were loaded at 30 µg per each well of 7.5% precast protein gels (Bio-Rad) and then separated by the sodium dodecyl sulfate polyacrylamide gel electrophoresis. The protein-separated gels were transferred electrically to PVDF membranes (Cosmo Bio, Tokyo, Japan). After the membranes were blocked with 5% skim milk and incubated with primary antibody to TLN1 (Bio-Rad) or β-actin (Sigma) followed by horseradish peroxidase-conjugated anti-mouse IgG secondary antibody (Abcam), the bands were visualized via chemiluminescence using ECL substrate kit (Merck) and ImageQuant LAS 4000 mini (Cytiva, Tokyo, Japan).

### Dual-luciferase reporter assay

HEK293T cells were seeded in a 96-well plate and allowed to grow to approximately 80% confluency. The cells were co-transfected with a pMirTarget-TLN1-3′ UTR vector (containing a firefly luciferase gene) (OriGene, Rockville, MD, USA), a pRL-SV40 vector (containing a Renilla luciferase gene) (Promega, Madison, WI, USA), and a pMIRNA1-miR-200c-3p mimic vector or pmiRZip antagomir vector by using Lipofectamine 2000 (Thermo Fisher Scientific). For control, cells were co-transfected with pMirTarget-TLN1-3′ UTR vector, pRL-SV40 vector, and a pSIH-H1 vector (System Biosciences). After 48 h of incubation, firefly and Renilla luciferase reporter activity were measured with a luminometer (2030 ARVO X4 multi label reader) using a Dual-Glo Luciferase Assay System (Promega) according to the manufacturer’s instructions. After normalizing reporter activities of firefly luciferase to those of Renilla luciferase, the ratio of luciferase activities was obtained.

### V-bottom well adhesion assay

Cells were fluorescently labeled with 1 mM 3′-O-acetyl-2′,7′-bis(carboxyethyl)-4 or 5-carboxyfluorescein, diacetoxymethyl ester (BCECF-AM) (Dojindo, Kumamoto, Japan). The cells were then washed with 4-(2-hydroxyethyl)-1-piperazineethanesulfonic acid (HEPES)-buffered saline (HBS) and counted. The wells of V-bottom 96-well plates (Greiner Bio-One GmbH, Frickenhausen, Germany) were coated with 10 µg/ml of fibronectin (Sigma-Aldrich) and incubated at room temperature for 90 min. The wells were then blocked with 2% BSA (Sigma-Aldrich) in HBS at 37 °C for 60 min and then washed twice with phosphate-buffered saline (PBS). Equal numbers of miR-200c-3p pmiRZip- or mimic/pre-miR-transduced cells or control vector-transduced cells resuspended in HBS were added to the well (2 × 10^5^/ml) with either 2 mM ethylenediaminetetraacetic acid (EDTA) (Wako) or 1 mM CaCl_2_ (Sigma-Aldrich) plus 1 mM MgCl_2_ (Sigma-Aldrich) and incubated at room temperature for 30 min. The samples treated with EDTA were used for estimating background adhesion to normalize integrin-mediated adhesion in the presence of Ca^2+^ and Mg^2+^^65^. The plates were centrifuged at 1500 rpm for 5 min to generate a shear stress and separate bound cells from unbound cells. Thus, this method can be suitable to calculate integrin-mediated cell adhesion, because the integrins usually undergo their conformational activation on cells in response to shear stress. After measurement of the fluorescence signal of the unbound cells by using a 2030 ARVO plate reader (PerkinElmer, Waltham, MA, USA), the cell adhesion to fibronectin was quantified as previously described^66^. Considering the post-centrifugal value of fluorescence in this assay was inversely proportional to percentage of the cells bound to fibronectin, the percentage of the bound cells was calculated by using the formula {100 – fluorescence (Ca^2+^/Mg^2+^) ÷ fluorescence (EDTA) × 100} as described previously with minor modifications^2,67^.

### Flat-bottom well adhesion assay

The adhesion assay using flat-bottom wells was performed to examine post-ligand binding as previously described^68,69^ with some modifications. The 96-well flat-bottom well plates (Thermo Fisher Scientific) were coated with fibronectin at 10 µg/ml and blocked with 2% BSA, followed by washes with PBS three times. As described above, the cells were fluorescently labeled with BCECF-AM. The cells were then incubated with either blocking antibody (5 µg/ml) to integrin β1 (Biolegend) and α5 (Biolegend) or mouse IgG1 (5 µg/ml, isotype control) (Biolegend) in the presence of Ca^2+^ (1 mM) and Mg^2+^ (1 mM) at room temperature for 30 min. After cells were added to the wells at 2 × 10^5^/ml, the plates were incubated at 37 °C for 60 min and washed three times gently with prewarmed PBS. The percentage of bound cells was calculated by measuring fluorescence with the 2030 ARVO plate reader before and after removal of unbound cells as described previously^69^.

### Flow cytometry

Antibody to activation-dependent epitope of β1 integrin (HUTS-4) was obtained from Sigma. Isotype control (mouse IgG2b) was purchased from Biolegend. The cells treated with HUTS-4 or isotype were fluorescently labeled by allophycocyanin-labeled anti-mouse IgG antibody, washed with PBS containing 2% FBS and 2 mM EDTA, and analyzed using BD Accuri C6 flow cytometer and software (BD Biosciences).

### Statistical analysis

Data are expressed as the mean ± standard errors of the mean (SEM). The two-tailed and unpaired Student’s t-test were used to compare two groups and one-way ANOVA was for more than two groups. *P* < 0.05 was considered significant. All assays were performed in more than triplicates and experiments were repeated at least three times. Statistical analysis was done with Prism 8 (GraphPad).

## Supplementary Information

Supplementary Figures.

Supplementary Tables.
