# Supplementary material for: miRNA-200c-3p targets talin-1 to regulate integrin-mediated cell adhesion
Source: Sci Rep. 2021 Nov 3;11:21597. doi: 10.1038/s41598-021-01143-3 (PMC8566560; doi:10.1038/s41598-021-01143-3)
Supplement: Supplementary file 1 — Supplementary Figures. [file 41598_2021_1143_MOESM1_ESM.pdf]

Supplementary information

miRNA-200c-3p targets talin-1 to regulate integrin-mediated cell adhesion

Gideon Obeng [1], Eun Jeong Park [1,#], Michael G. Appiah [1], Eiji Kawamoto [1,2], Arong Gaowa [1], and Motomu Shimaoka [1,#]

[1]Department of Molecular Pathobiology and Cell Adhesion Biology, Mie University Graduate School of Medicine, Tsu, Mie 514-8507, Japan
[2]Department of Emergency and Disaster Medicine, Mie University Graduate School of Medicine, Tsu, Mie 514-8507, Japan

Running title: miR-200c-3p coordinates integrin-mediated cell adhesion

[#]Correspondence: Eun Jeong Park, Department of Molecular Pathobiology and Cell Adhesion Biology, Mie University Graduate School of Medicine, Tsu, Mie 514-8507, Japan; Tel: +81-59-231-6408; Email: epark@med.mie-u.ac.jp; Motomu Shimaoka, Department of Molecular Pathobiology and Cell Adhesion Biology, Mie University Graduate School of Medicine, Tsu, Mie 514-8507, Japan; Tel: +81-59-231-5036; Email: shimaoka@med.mie-u.ac.jp

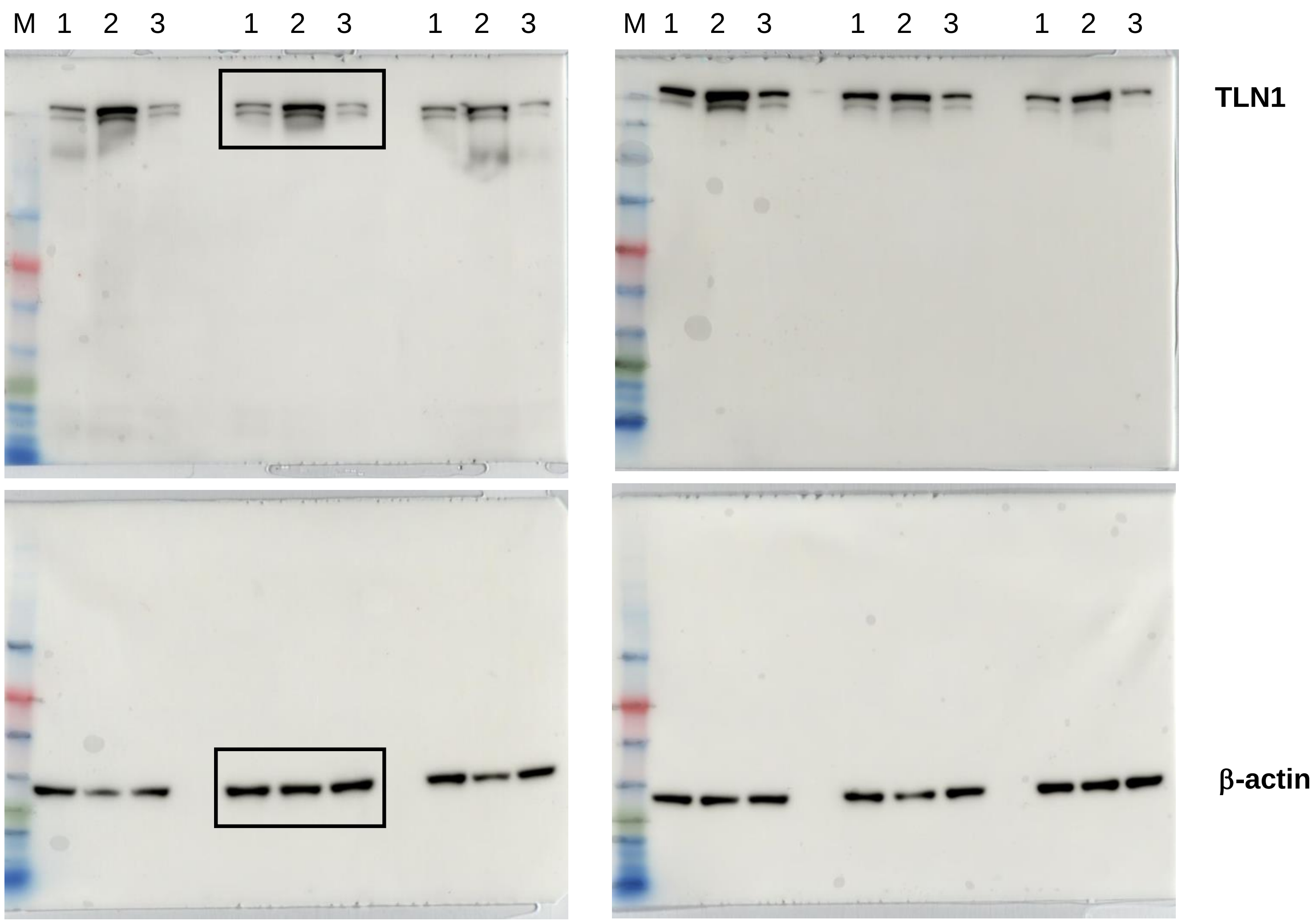

Figure S1. Immunoblot scans. Immunoblots of TLN1 and β-actin are shown in upper and lower panels, respectively. Boxes indicate the bands for the Figure 2F. The same amounts of lysates samples derived from the cells transduced with cont. vec., miR-200c-3p pmiRZip, and miR-200c-3p mimic, were applied to lanes 1, 2, and 3, respectively. Two independent experiments (left and right panels) using three different samples per blot were performed. M, protein size marker.

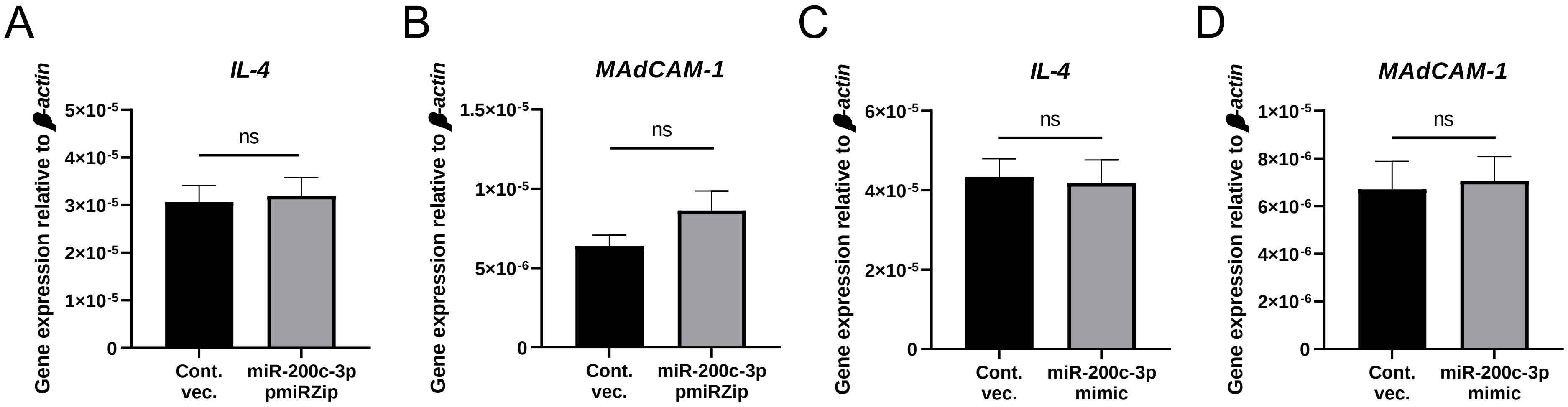

Figure S2. IL-4 and MAdCAM-1 gene expressions in the miR-200c-3p pmiRZip and miR-200c-3p mimic clones. Expressions of non miR-200c-3p-target genes (*IL-4* and *MAdCAM-1*) in clones for miR-200c-3p pmiRZip (A&B) and miR-200c mimic (C&D) transduced cells were analyzed in comparison with control vector-transduced cells. The RNA samples extracted from the HEK293T cell clones were subjected to the gene-expression analysis with RT-qPCR. Relative expression to a reference gene (*β-actin*) using comparative threshold (CT) values was shown as $2^{-\Delta CT}$. All assays were performed in triplicates and the experiments were repeated three times. Data are expressed as the mean ± standard errors of the mean (SEM). Cont. vec., control vector; IL-4, interleukin 4; MAdCAM-1, mucosal addressin cell adhesion molecule 1; and ns, not significant.

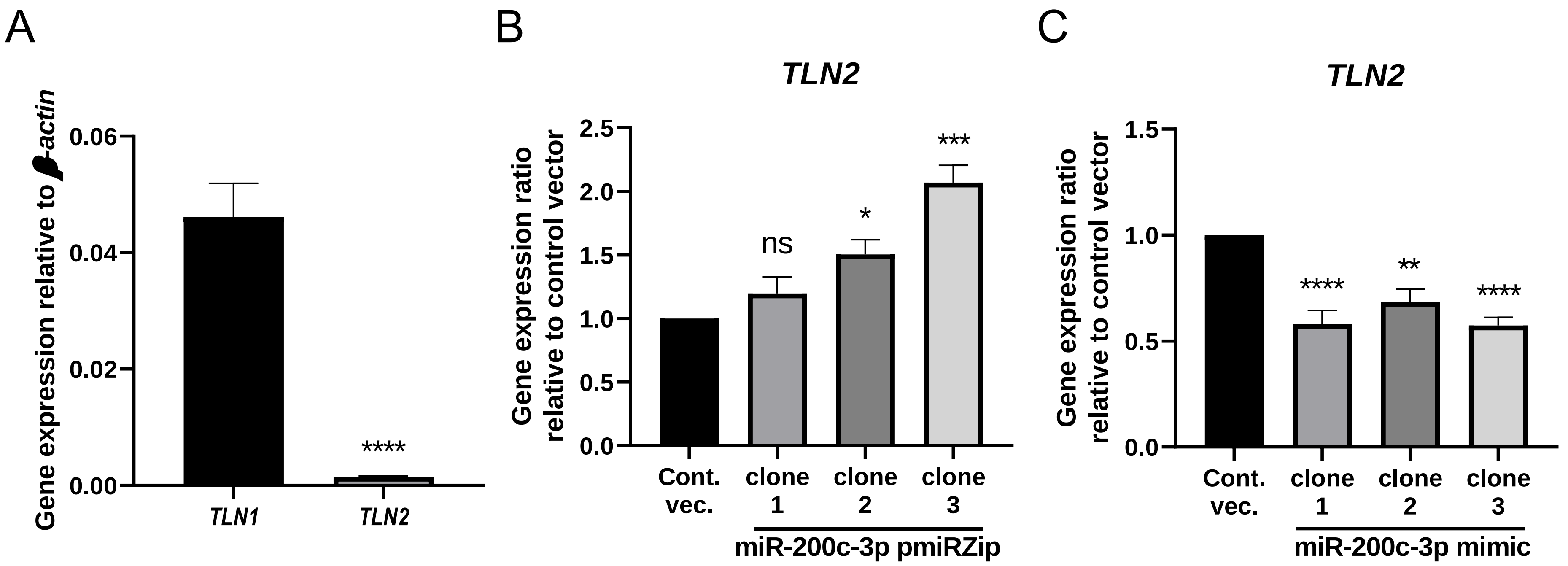

Figure S3. Expressions of *TLN2* in HEK293T cells. (**A**) Relative expression of endogenous *TLN1* and *TLN2* to the reference gene (β-*actin*) in native HEK293T cells using comparative threshold (CT) values was shown as $2^{-\Delta CT}$. (**B**&**C**) HEK293T cells were stably transduced with miR-200c-3p pmiRZip, miR-200c-3p mimic, and empty vector (control) using lentivector systems. The expression levels of *TLN2* in different clones for either KD (pmiRZip) (**B**) or mimic/pre-miR (**C**) were measured using RT-qPCR. Data are expressed as the mean ± standard errors of the mean (SEM). Cont. vec., control vector; and ns, not significant. * P < 0.05; ** P < 0.01; *** P < 0.001; and **** P < 0.0001 relative to the *TLN1* (**A**) or control vector (**B**&**C**).

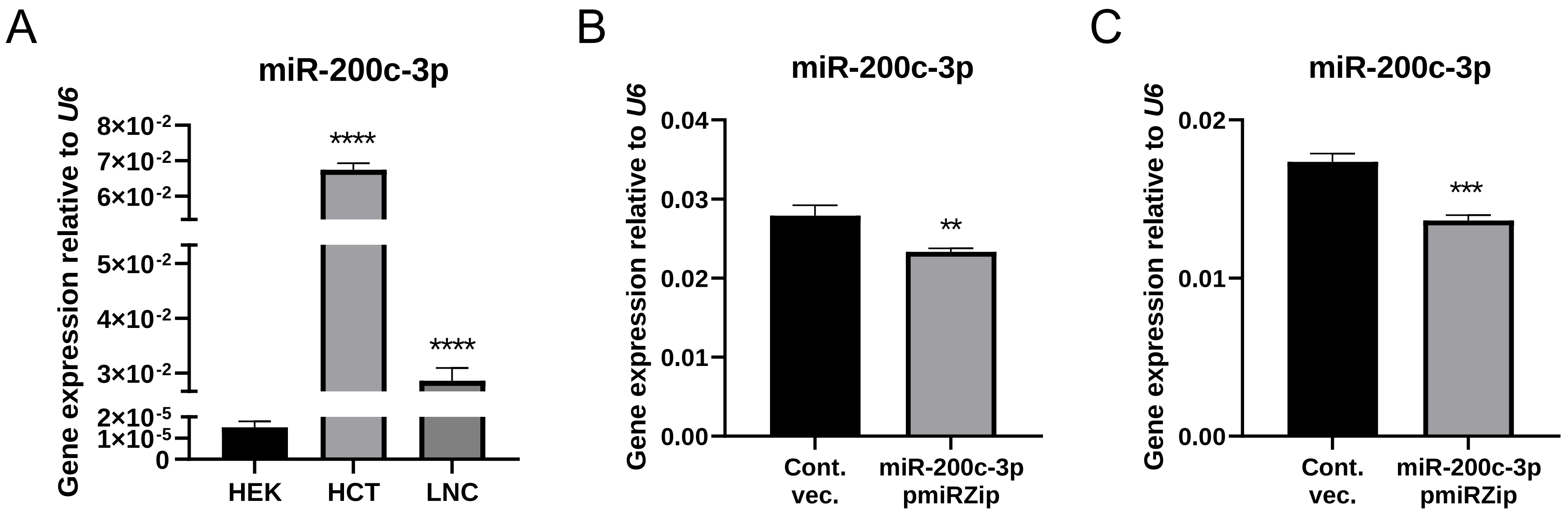

Figure S4. Comparison of miR-200c-3p levels in different cell lines. (**A**) The endogenous levels of miR-200c measured by RT-qPCR were compared between native untreated HEK293T, HCT116, and LNCaP cells. (**B**&**C**) HCT116 (**B**) and LNCaP cells (**C**) were transduced with either control or miR-200c-3p knockdown (pmiRZip) vectors using lentivector system and miR-200c-3p expressions were measured using RT-qPCR. (**A**-**C**) Relative expression to the reference gene (*U6*) using comparative threshold (CT) values was shown as $2^{-\Delta CT}$. All assays were performed in six wells and the experiments were repeated three times. Data are expressed as the mean ± standard errors of the mean (SEM). HEK, HEK293T; HCT, HCT116; LNC, LNCaP; cont. vec., control vector. ** $P < 0.01$; *** $P < 0.001$, and **** $P < 0.0001$ relative to the HEK (**A**) or control vector (**B**&**C**).

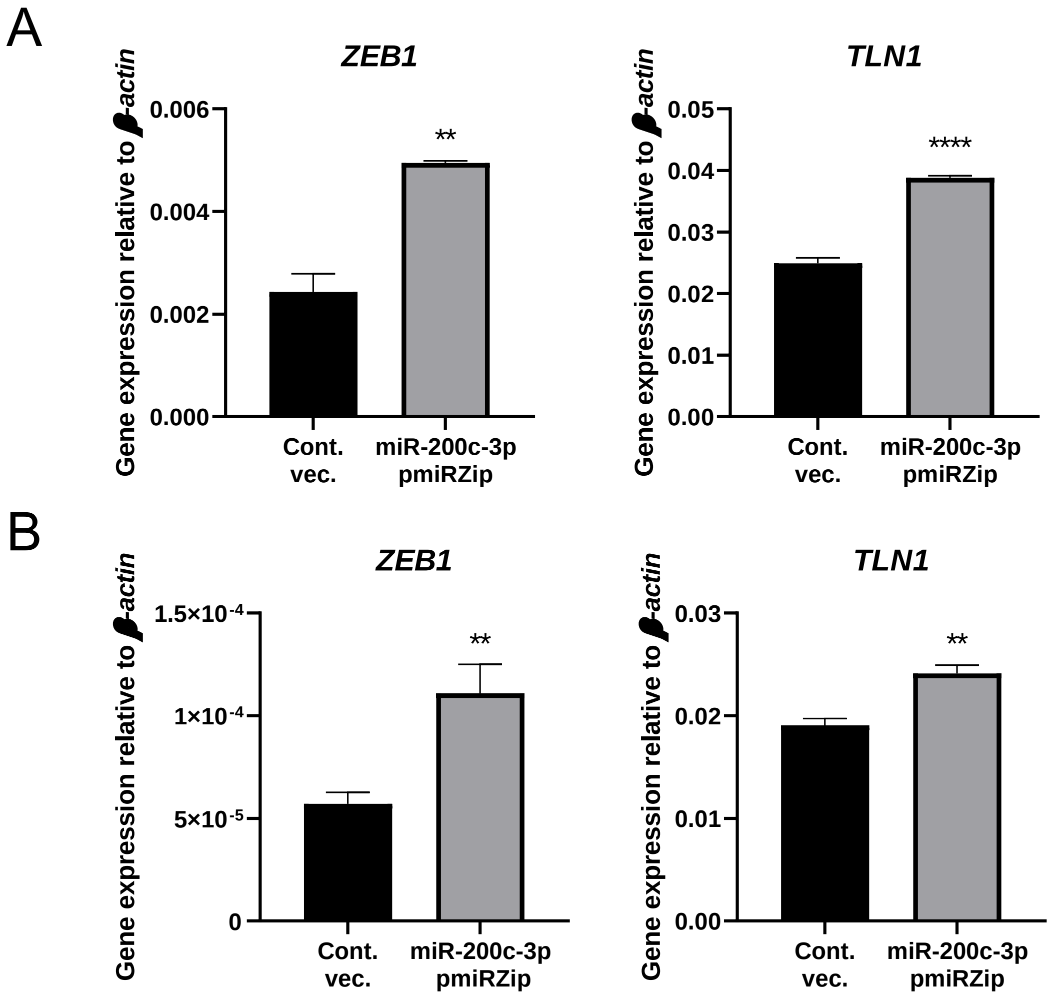

Figure S5. Expressions of *ZEB1 and TLN1* in HCT116 and LNCaP transfectants. (A&B) HCT116 (**A**) and LNCaP (**B**) cells were transduced with either control or miR-200c-3p pmiRZip using lentivector system. The expression levels of *ZEB1* mRNA (left panel) and *TLN1* mRNA (right panel) were measured using RT-qPCR. Relative expression to the reference gene (*β-actin*) using comparative threshold (CT) values was shown as $2^{-\Delta CT}$. All assays were performed in six wells and the experiments were repeated three times. Data are expressed as the mean ± standard errors of the mean (SEM). Cont. vec., control vector. ** $P < 0.01$; and **** $P < 0.0001$ relative to the control vector.

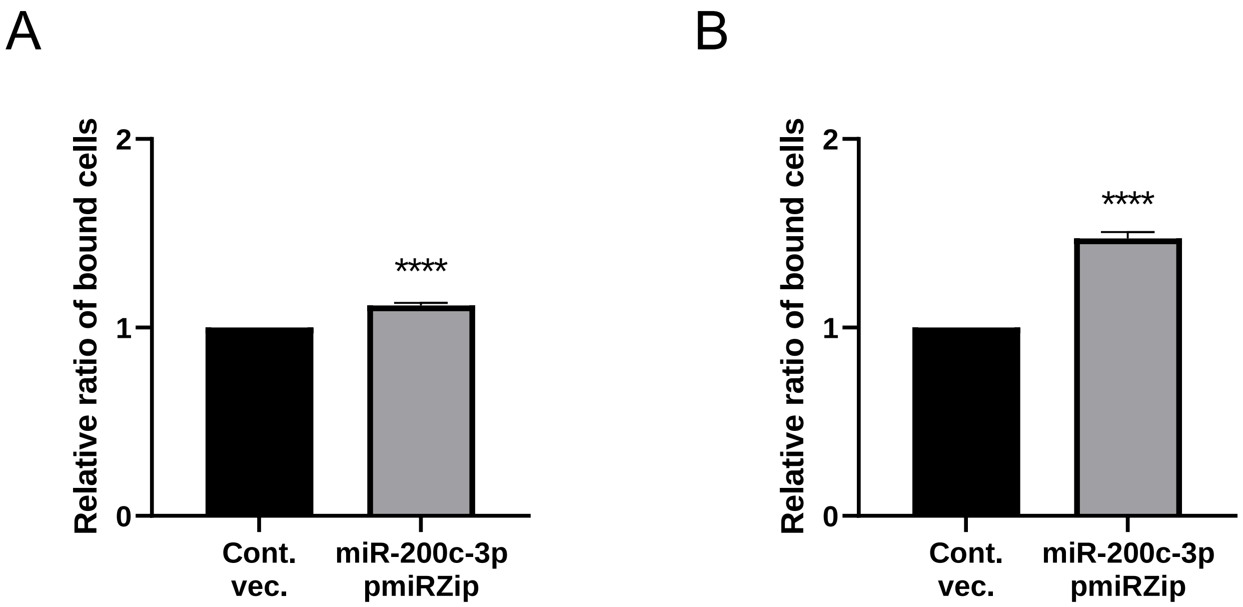

Figure S6. Knockdown of miR-200c-3p enhanced cell binding to fibronectin. (A&B) Adhesion of HCT116 (**A**) and LNCaP (**B**) cells to fibronectin substrates were studied using a V-bottom well plate-based assay using the cells transduced with either control or miRNA-200c-3p pmiRZip vectors. The percentages of bound cells were determined as described in the Methods section, and the relative ratios to control vector-transduced cells are shown. All assays were performed in six wells and the experiments were repeated three times. Data are expressed as the mean ± standard errors of the mean (SEM). Cont. vec., control vector. **** $P < 0.0001$ relative to the control vector.

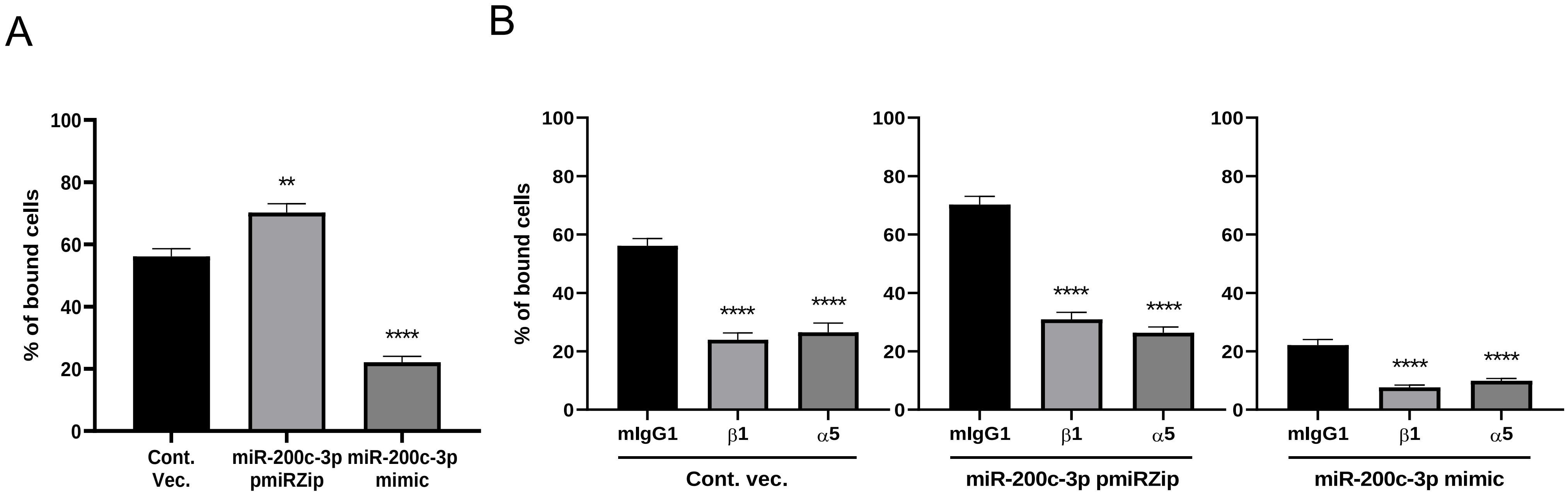

Figure S7. miR-200c-3p regulates α5β1-induced cell binding to fibronectin. (**A**) Percentage of bound cells to flat-bottom wells coated with fibronectin was measured for those cells transduced with control vector, miR-200c-3p pmiRZip, and miR-200c-3p mimic vector. (**B**) Effects of pretreatment of blocking antibodies (integrins β1 and α5) on affecting cell binding to these flat-bottom wells were examined in those cell clones indicated. All assays were performed in more than triplicates and the experiments were repeated three times. Data are expressed as the mean ± standard errors of the mean (SEM). Cont. vec., control vector; mIgG1, mouse IgG1 isotype control; β1, anti-β1 antibody; and α5, anti-α5 antibody. ** $P < 0.01$; and **** $P < 0.0001$ relative to the control vector (**A**) or mIgG1 (**B**).

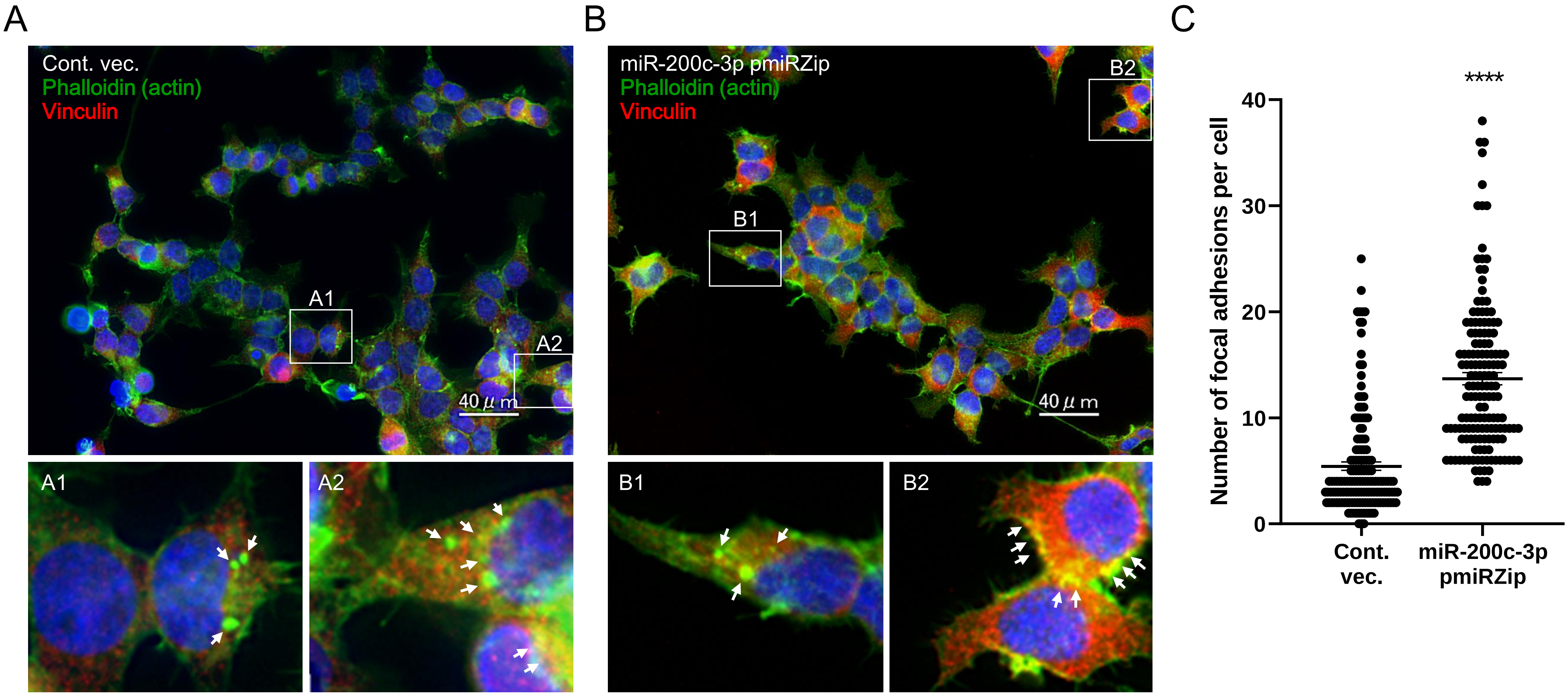

Figure S8. The cells stably transduced with miR-200c-3p pmiRZip enhance focal adhesion formation. (A&B) Representative immunofluorescence images for staining phalloidin (green) and vinculin (red) are shown in the HEK293T cells transduced with control vector (**A**) or miR-200c-3p miRZip (**B**). A few representative cells boxed inside the upper image were magnified and blow-up images were added below (**A1**, **A2**, **B1**, & **B2**). These blow-up images and arrows show which spots are counted as focal adhesion plaques. Nuclear staining was done with DAPI (blue). Scale bar, 40 μm. (**C**) A scatter plot comparing the number of focal adhesions per cell. Horizontal thick and thin lines overlayed on dots denote the mean and standard errors of the mean (SEM), respectively. More than one hundred fifty cells from three different images per group (see Figure S9 for more detail) were used to manually quantify focal adhesions. Cont. vec., control vector. **** $P < 0.0001$ relative to the control vector (**C**).

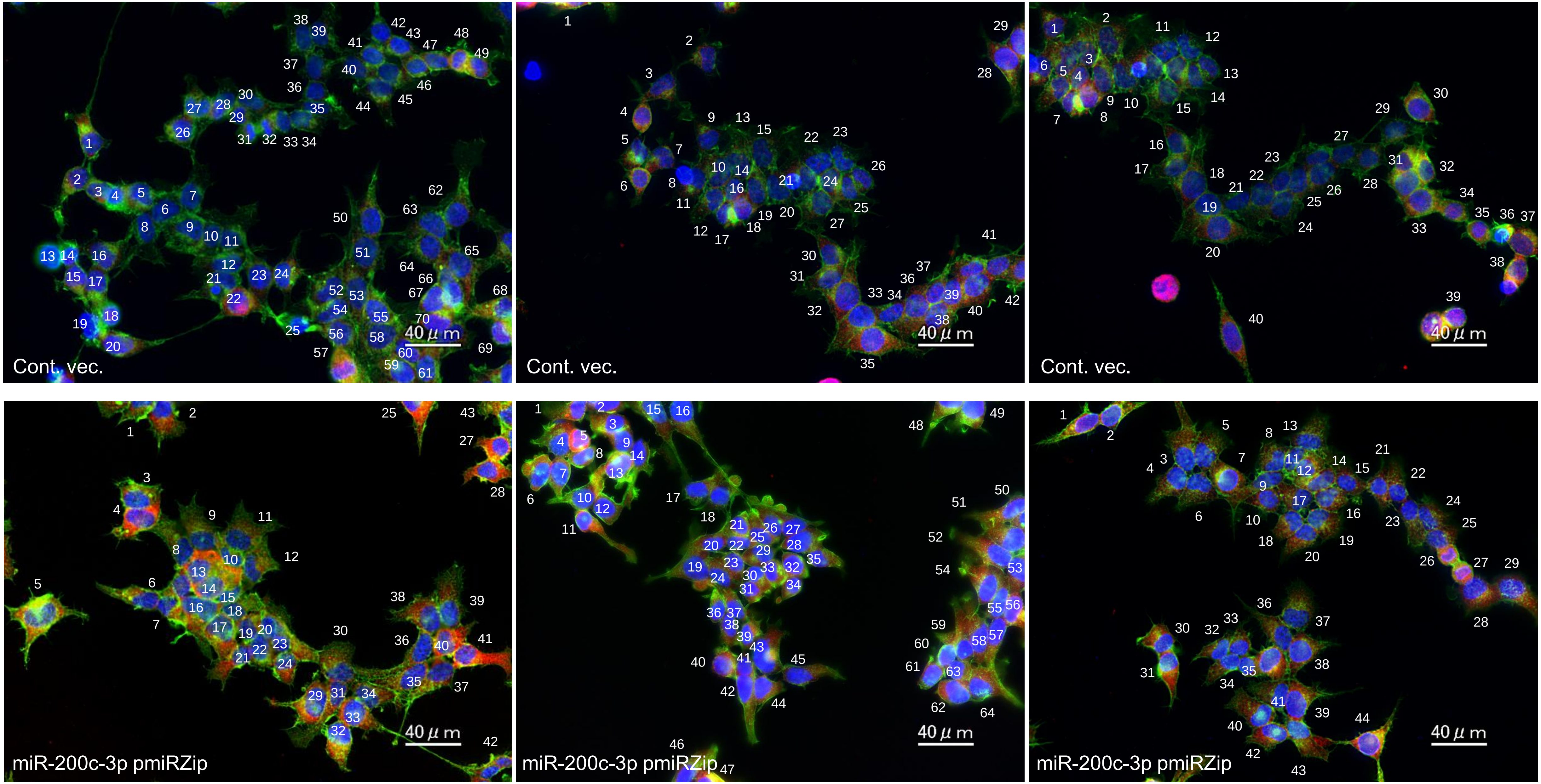

Figure S9. Three representative images per group were used in quantifying focal adhesions for the scatter plots shown in Figure S8C. Each cell numbers are shown in images. Scale bar, 40 μm. Cont. vec., control vector.
